# Addressing the state of surgical care In Kenya: challenges, opportunities, and future directions

**DOI:** 10.1097/MS9.0000000000002667

**Published:** 2024-10-16

**Authors:** Piel P. Kuol, Peter N. Wambu, Ruth W. Mwangi, Bartay Matui, Alvin Kiprop, Kevin S. Sokoto, Emmanuel Gudu

**Affiliations:** School of Medicine, Moi University, Eldoret, Kenya

## Introduction

Access to safe, prompt, and cost-effective surgical care is a fundamental right essential for human health. It is estimated that an additional 17.5 million surgical procedures are necessary each year to address the surgical demands in Eastern sub-Saharan Africa. Nonetheless, the actual figure is likely to be higher, as existing estimates often underestimate the true requirements when extrapolated across various nations^1^. In Kenya, a lower-middle-income economy with a population of 52.5 million, access to surgical care is a pivotal mechanism for enhancing the overall quality of healthcare^2^. This analysis delves into Kenya’s current status and advancements on six critical indicators, alongside the objectives established by national policies. Furthermore, it addresses many challenges facing the attainment of equitable and sustainable surgical practices within the nation’s decentralized healthcare system.

The 2015 Lancet Commission on Global Surgery (LCoGS) delineated six pivotal indicators with specified targets to denote robust national surgical systems^2^. These indicators, elucidated alongside their 2030 global objectives, underscore indispensable surgical infrastructure, workforce, and outcomes and have been endorsed by the WHO as part of a comprehensive surgical reporting schema^3^. The indicators are segmented into three categories: preoperative, perioperative, and postoperative.

The preoperative objectives are bifurcated: ensuring 80% of the populace resides within a 2 h radius of a hospital proficient in performing the three essential procedures (laparotomy, cesarean delivery, and open fracture care), and attaining 20 surgery, anesthesia, and obstetrics (SAO) practitioners per 100 000 inhabitants. The perioperative objectives encompass executing 5000 surgical interventions per 100 000 inhabitants annually and instituting a national mechanism to monitor the perioperative mortality rate (POMR) and surgical volume. The postoperative objectives involve 100% safeguarding against impoverishing expenditures (IE) and catastrophic healthcare expenditures (CHE) consequent to surgical interventions.

Since independence in 1963, Kenya’s healthcare delivery has undergone various reforms^4^, the most significant being the recent devolution of service delivery and management to 47 county governments. The central Ministry of Health collaborates with local authorities to manage funds and resources and develop national health policies^5^. The Kenya Essential Packages for Health has introduced a six-tiered healthcare delivery framework, ranging from local community health units to national referral hospitals, with each facility’s classification determining its service expectations^6^.

These reforms align with the global recognition that district hospitals, alongside national hospitals, should play a crucial role in providing accessible surgical care^7^. Devolution enhances decision-making authority over human resources and financial oversight. However, disparities in surgical service access persist within and between counties^5,8^. This review explores how the LCoGS indicators can evaluate surgical capacity progress in a low-income country and middle-income country, focusing on Kenya’s surgical system and incorporating local policies into a national surgical capacity assessment.

## Current challenges

The inadequacy of surgeons^9^ in Kenya routes to training individuals from their basic healthcare programs to the training of accomplished doctors to various surgery programs^10^. A wider input of medical students into medical universities is met with a bottleneck in graduating the same students. Career choice for the graduates also differs^11^. However, the training facilities for surgical students in different fields are limited by the few hospital and school facilities in their selected fields. It should also be noted that training of medical students and surgeons is very expensive. This stems from the availability of tutors, availability of learning materials, accommodation, and learning equipment. The availability of funding from the government fluctuates with the government’s budget for the health sector and this deeply blows the idea of flawless training^9^. The ultimate juggernaut in the surgeon shortage in Kenya is the high levels of brain drain^12^. Trained surgeons seek to study and work abroad, in a bid to seek greener pastures, better work-life balance, broader exposure to the kaleidoscope of health provision, and greater demand in the developed nations, hence better incentives^9^. It is also noted that trained undergraduates also seek to study overseas and this reduces the expected influx of personnel in the specialist field, including the surgical field^9,11^

There is an imbalance in the access to surgical care in Kenya^13^. The effect of this is highly catapulted by the devolution of healthcare^8^. This oversees the setting up of surgical services in levels 4, 5, and 6 that are widely distributed in the urban areas with only 30 and 2% of tertiary and primary healthcare facilities being able to provide both a surgical and anesthetist service. Therefore, the lower levels, which are popular in rural areas have little to no surgical attention despite the caseloads experienced in the area^14^. This coupled with the few medical resources in rural areas quickly couples the state of surgical care in the rural areas^13^.

The imbalanced resource distribution in rural areas has many grave effects, including high mortality rates.^15^ This is caused by the untimely or inadequate operative care on cases that much needed the same. Underdeveloped infrastructural capacities such as roads also deeply incapacitate the slight effects of seeking healthcare from the nearest facility. Some of the cases include trauma, appendectomy, and neurosurgical care^14^, which, when performed by untrained personnel, compromises outcome^16^. Delayed treatment in such cases due to surgeon shortage also exacerbates the magnitude of the cases. This leads to a deteriorated quality of life, life-threatening effects, and also permanent disability. In other cases, an increased socioeconomic burden is experienced when the patients move to seek better healthcare^17^. Transport, accommodation, and surgical fees coupled with the high poverty burden in rural areas greatly affect the patient^8^.

## Opportunities for improvement

### Prioritizing incentive programs

The uneven distribution of surgeons in urban areas compared to rural areas is a hefty challenge to the improvement of surgical care in the latter areas. To address this disparity, it is imperative to delve into the potential of financial and career development incentives to attract surgeons to rural areas. Financial incentives would prove to be vital as offering competitive salaries, housing allowances, and relocation packages can make rural postings more attractive to surgical healthcare providers^18^.

Career development incentives through establishing clear career advancement pathways for surgeons working in rural areas are crucial. This could include opportunities for further training, research grants, and the possibility of fast-tracking promotions^19^.

### Medical education and training

The creation and advancement of more medical schools and greater capacity at already existing ones can aid in the production of surgeons. The Kenyan healthcare system will undergo a mindset shift toward a greater emphasis on rural healthcare by adding additional residency and fellowship slots. Creating more residency and fellowship positions based and centered on rural healthcare will provide a mental shift in the Kenyan healthcare system to be inclined toward rural healthcare. There needs to be a regular review of training curriculums to incorporate the latest advancements. A focus on experiential, hands-on learning, and simulation-based education can better prepare medical residents and students for the surgical challenges they will face in the real world^18^.

### International partnerships

Kenyan surgeons and medical students can potentially be exposed to cutting-edge surgical procedures and best practices by establishing exchange programs with foreign medical schools and hospitals. This would increase its capability for medical research through initiatives including working on joint research projects with foreign institutions.

Collaborative projects can concentrate on topics that both parties find interesting, like tropical illnesses, public health, and cutting-edge surgical methods. This helps with local health issues as well as adding to the corpus of medical knowledge worldwide^20^.

Training workshops and seminars: Partnering with international organizations to host training workshops and seminars in Kenya can bring world-class expertise directly to local surgeons. These events can cover a range of topics, from new surgical technologies to patient care standards^18^.

Resource sharing: International partnerships can facilitate the donation of medical equipment and supplies, as well as the sharing of digital resources such as medical journals, online courses, and telemedicine services. This can help bridge the resource gap and ensure that Kenyan surgeons have access to the tools they need to provide high-quality care^20^.

## Future directions

The following points should be considered for improving access to surgical care in rural areas.

(1). Policymakers should focus on extending health insurance coverage to achieve universal health coverage.^21^ This can be done by creating tailored insurance programs for individuals with irregular incomes, ensuring efficient premium collection and risk-pooling, and allocating a portion of taxes to public health insurance programs. Public health campaigns should also be conducted to educate the informal sector on the importance of health insurance.

(2). Upgrading existing facilities and creating specialized surgical units in county-level and sub-county-level hospitals^22^ will provide better access to high-quality medical services in rural areas. This includes providing education programs and workshops for rural healthcare professionals, advanced diagnostic tools, modern surgical instruments, and maintaining hygienic standards.

(3). Utilizing telemedicine and mobile health units can significantly contribute to providing services in rural areas by enabling early diagnosis, improving symptom management, and preventing postoperative complications.^23^ This can be done through video or mobile consultation, mobile applications, data trackers, interactive websites, and consultations with specialists worldwide.

## Conclusion

Addressing the state of surgical care in Kenya requires a comprehensive and strategic approach which includes policy reforms, infrastructure development, and international partnerships. The need for focused efforts is highlighted by the challenges seen in the uneven distribution of surgeons, inadequate surgical training, and limited resources in rural regions.

The goal of achieving universal health coverage is nearly impossible without expanding health insurance coverage as financial constraints remain a major barrier to healthcare access. Moreover, the quality and reach of surgical care can be enhanced by upgrading facilities in underserved rural areas.

To attract and retain surgeons in rural areas and the country, it is essential to implement financial incentives and career development programs. Additionally, Kenyan surgeons will greatly benefit from international partnerships that provide exposure to the latest surgical techniques and best practices ultimately improving patient care and outcomes.

By implementing these strategies, Kenya can make significant progress in providing surgical care to its citizens. These will not only improve health outcomes but also enhance the overall quality of life for the population.

## Ethical approval

Ethics approval was not required for this require for this review.

## Consent

Informed consent was not required for this review.

## Source of funding

None.

## Author contribution

P.P.K.: conceptualization; P.P.K., P.N.W., B.M., and R.W.M E.G: writing – original draft; P.P.K., B.M., and K.S.S.: writing – review and editing.

## Conflicts of interest disclosure

The authors declare no conflicts of interest.

## Research registration unique identifying number (UIN)

Not applicable to this review.

## Guarantor

Piel Panther Kuol.

## Data availability statement

Not applicable for this review.

## Provenance and peer review

Not commissioned, externally peer-reviewed.

## Assistance with the study

None.
